# Genetic Evidence of Importation of Drug-Resistant *Plasmodium falciparum* to Guatemala from the Democratic Republic of the Congo

**DOI:** 10.3201/eid2006.131204

**Published:** 2014-06

**Authors:** Jaymin C. Patel, Steve M. Taylor, Patricia C. Juliao, Christian M. Parobek, Mark Janko, Luis Demetrio Gonzalez, Lucia Ortiz, Norma Padilla, Antoinette K. Tshefu, Michael Emch, Venkatachalam Udhayakumar, Kim Lindblade, Steven R. Meshnick

**Affiliations:** University of North Carolina, Chapel Hill, North Carolina, USA (J.C. Patel, S.M. Taylor, C.M. Parobek, M. Janko, M. Emch, S.R. Meshnick);; Centers for Disease Control and Prevention, Atlanta, Georgia, USA (P.C. Juliao, V. Udhayakumar, K. Lindblade);; Military Medical Center, Guatemala City, Guatemala (L.D. Gonzalez);; Universidad de Valle de Guatemala, Guatemala City (L. Ortiz, N. Padilla);; University of Kinshasa, Kinshasa, Democratic Republic of the Congo (A.K. Tshefu)

**Keywords:** Malaria, *Plasmodium falciparum*, protozoan, parasite, Anopheles mosquito, microsatellites, molecular genotyping, drug-resistant, chloroquine phosphate, antimalarial, Guatemala, Democratic Republic of the Congo

## Abstract

Imported malaria threatens control and elimination efforts in countries that have low rates of transmission. In 2010, an outbreak of *Plasmodium falciparum* malaria was reported among United Nations peacekeeping soldiers from Guatemala who had recently returned from the Democratic Republic of the Congo (DRC). Epidemiologic evidence suggested that the soldiers were infected in the DRC, but local transmission could not be ruled out in all cases. We used population genetic analyses of neutral microsatellites to determine the outbreak source. Genetic relatedness was compared among parasites found in samples from the soldiers and parasite populations collected in the DRC and Guatemala; parasites identified in the soldiers were more closely related to those from the DRC. A phylogenetic clustering analysis confirms this identification with >99.9% confidence. Thus, results support the hypothesis that the soldiers likely imported malaria from the DRC. This study demonstrates the utility of molecular genotyping in outbreak investigations.

## Abstract 2

Molecular markers and population genetics were effective tracking tools.

Imported malaria threatens control and elimination efforts in countries that report low malaria transmission rates (*1*–*3*). In Central America, malaria transmission decreased by >50% during 2000–2010 (*4*); in 2010, the Guatemala Ministerio de Salud Pública y Asistencia Social reported 31 confirmed cases of malaria, all caused by the species *Plasmodium falciparum* (*5*). Central America is unusual compared with other areas in which malaria is endemic because chloroquine remains an effective treatment option for *P. falciparum* infection there, but not in other parts of the world (*6*–*8*); the introduction of parasites harboring chloroquine-resistant genotypes could fuel a resurgence of clinical illness and transmission.

In 2010, an outbreak of malaria was reported among 12 soldiers from Guatemala shortly after they returned from a United Nations (UN) peacekeeping mission in the Democratic Republic of the Congo (DRC). Of the 12, 8 also reported visiting >1 area in Guatemala in which malaria is endemic when they returned but before the outbreak was identified. An outbreak investigation was undertaken after 1 of the infected soldiers died; laboratory tests of blood from this patient identified choroquine-resistant and -sensitive strains of *P. falciparum*.

The epidemiologic evidence suggested that the soldiers were infected while stationed in the DRC (*9*). Because the local acquisition of chloroquine-resistant parasites in Guatemala could necessitate a change in local treatment practices, it was vital to determine the origin of the soldiers’ infections.

Molecular markers have been used to assess the genetic relatedness of malarial parasites from different geographic regions (*10*,*11*). Accordingly, if the soldiers acquired *P. falciparum* in the DRC during their stay, the genotypes of the parasites isolated from the soldiers’ samples would be more closely related to parasites from the DRC than to parasites from Guatemala. To test this hypothesis, we used molecular methods from the field of population genetics to determine the source of the malaria outbreak among the soldiers who returned to Guatemala after being stationed in the DRC.

## Methods

### Study Participants

We included *P. falciparum* parasites from 3 distinct populations: 1) soldiers from Guatemala returning from the DRC with malaria; 2) adult residents of the DRC; and 3) residents of Guatemala (adults and children). The initial outbreak investigation in Guatemala received appropriate human subject review by the Universidad del Valle de Guatemala (Guatemala City, Guatemala) and the US Centers for Disease Control and Prevention (Atlanta, GA, USA) and was qualified as public health practice because its purpose was to identify and treat malaria cases among military personnel returning from the DRC. Samples from these soldiers were anonymized, and the investigations reported in this publication were reviewed and approved by the same institutions. Samples from the DRC were obtained during the 2007 Demographic Health Survey (DHS), which was approved by the review boards of Macro International (Calverton, MD, USA), the University of Kinshasa School of Public Health (Kinshasa, DRC), and the University of North Carolina (Chapel Hill, NC, USA). Samples collected from a previous malaria surveillance study (conducted during 1998–2000) that was originally approved by the Universidad del Valle de Guatemala human subjects review board were used to determine population structure of the parasite population of Guatemala.

In August of 2013, Guatemala sent its 13th mission to the DRC since it began sending troops in 2000 (*12*). In January 2010, 144 soldiers from Guatemala and 6 civilian support staff were deployed to the DRC as part of a United Nations peacekeeping mission. Upon return to Guatemala in October 2010, 12 soldiers were found to be infected with *P. falciparum* by using active and passive case detection; the infections were confirmed by using nested PCR. Of the 12 soldiers, 5 reported clinical symptoms and the other 7 were asymptomatic; date of onset of symptoms for the 5 soldiers ranged from October 12, 2010 (5 days before leaving the DRC) to November 7, 2010 (3 weeks after their arrival in Guatemala) (*9*). The first soldier in whom malaria was diagnosed died and was found to have been infected with parasites that had chloroquine-sensitive and chloroquine-resistant genotypes (*9*). Samples from all 12 soldiers were included in the analysis reported here.

**Figure 1:**
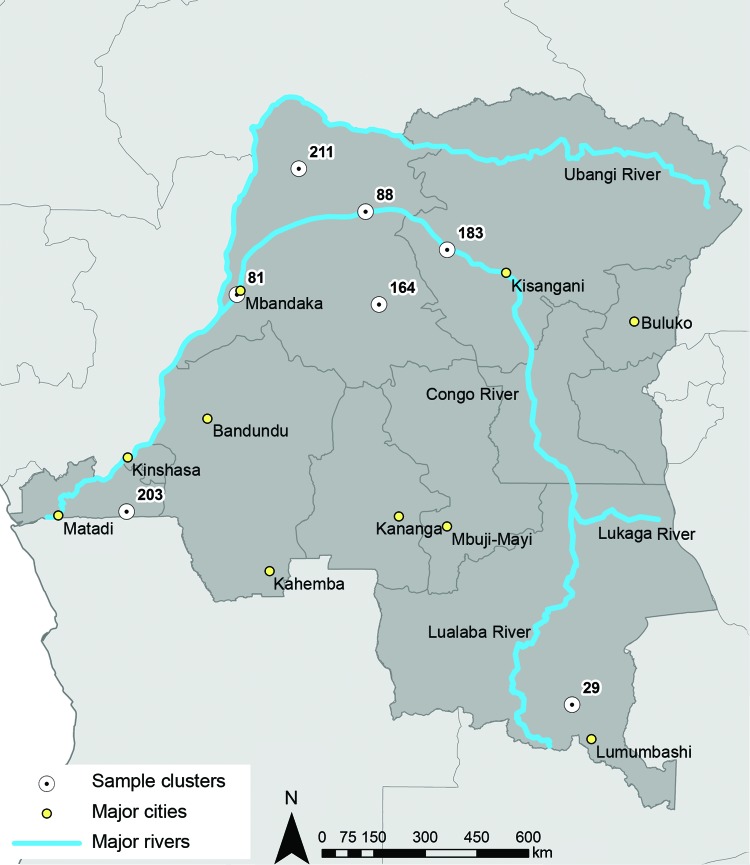
Location of the major cities, rivers, and the 7 Demographic Health Survey clusters (203, 81, 88, 183, 211, 164, and 29) within the Democratic Republic of the Congo (DRC) included in study of malaria outbreak among soldiers from Guatemala who had been stationed in DRC.

To compare the parasites found in the DRC with those that infected the returning soldiers, we analyzed *P. falciparum* parasites from 74 participants in the national 2007 DRC DHS. The parent study and ancillary studies have been described in detail (*13*–*16*). For the analysis reported here, we selected 7 clusters, all of which included >10 *P. falciparum*–positive persons, as part of a study to quantify gene flow in *P. falciparum* strains within the DRC. Three outbreak clusters (81, 88, and 183) were chosen because they were located on or near the Congo River, a principal route of human transportation (*17*); 2 clusters (164 and 211) were chosen because they were not on the river but were approximately the same distance apart from the 3 river sites as the river sites were from each other; and 2 clusters (29 and 203) were chosen because they were far away from the other clusters (Patel et al., unpub data) (Figure 1). We also included 40 *P. falciparum* specimens collected from previous surveillance studies conducted in Guatemala during 1998–2000 for comparison of genetic profiles of the local parasite population.

### Microsatellite Analysis 

Genomic DNA (gDNA) from the DRC samples was isolated from dried blood spots as described (*13*). We used the QIAamp DNA Mini Kit (QIAGEN, Valencia, CA, USA) according to the manufacturer’s recommendations to extract gDNA from the soldiers and from samples from persons who were indigenous to Guatemala. All samples were sealed securely and stored at −20°C.

Eight neutral microsatellites were selected on chromosomes 2 (C2M33, C2M34, C2M29, C2M27) and 3 (C3M40, C3M88, C3M39, and C3M69) to assess whether the parasites identified in the soldiers were related to those found in the DRC. PCR primer sequences and cycling conditions for samples from the soldiers and other persons in Guatemala were adapted from earlier studies (*18*). For the samples from the DRC, a slightly different PCR technique was used. Specimens were initially amplified in single-round PCR protocols similar to those for samples from the soldiers and from persons in Guatemala; for those that failed to return PCR products, we used a heminested strategy wherein a newly designed additional external primer was used in a primary amplification, and then performed the standard round of amplification (*19*,*20*). Primer sequences are provided in the Table; their PCR cycling parameters have been described elsewhere (*18*,*21*). All PCR products were separated by capillary electrophoresis by using an Applied Biosystems 3130xl genetic analyzer (Applied Biosystems, Foster City, CA). The alleles were scored by using GeneMapper software, version 3.7 (Applied Biosystems). Alleles were binned to the nearest 2 or 3 nucleotides in length depending on the size of the repeat unit. To distinguish alleles from background noise in multiple infections, we recorded peaks if they were >1/3 of the maximum peak level and exceeded 100 fluorescence units.

**Table:** Neutral microsatellite loci and primer sequences used for PCR amplification of *Plasmodium* f*alciparum* malaria genes to identify origin of drug-resistant genotype, Guatemala

Locus name	Chromosome	Primer	Primer sequence (5′→3′)	Tag	Expected product size, bp
C2M33	2	Forward	CATTGCAAAATATATATTCTCC	FAM	193
		Reverse	GTGATTTGTACAATGTACATA		
		Heminested	ATTGCGTAAATAACACATCTGCA		
C3M88*	3	Forward	CAAAAATGAAAAATGAAAAGG	HEX	150
		Reverse	TAAAGGGTGCGCATATCAAT		
		Heminested	GTTATTCAAAAAGGACGAAACAAG		
C3M69	3	Forward	AATAGGAACAAATCATATTG	HEX	173
		Reverse	AGATATCCAGGTAATAAAAAG		
		Heminested	TTTATGAACACCCTCATGTCACT		
C2M29	2	Forward	GTGAATAACGGAAAAGGATA	FAM	141
		Reverse	AAGATCAAATACCAGGTGA		
		Heminested	TTAAGAAACAATCAGAAGCGATG		
C2M27	2	Forward	CTTTTAATCACTACCATGTTG	HEX	117
		Reverse	ATAATTTAATTGAGGATACCT		
		Heminested	TTGTATGTATCACTTTTTCATTAC		
C3M40	3	Forward	GGGTAAAGAAAAACACACAAA	FAM	128
		Reverse	AATGTGTATATTACTAGAAGC		
		Heminested	TCCGAATATGGAATGTCGAAAG		
C3M39	3	Forward	CAAGAAGATAGGGATGATAAC	FAM	159
		Reverse	TATTAATTGGTCTTCACCCG		
		Heminested	GGAGGAACGTAAAGAAGATATTG		
C2M34	2	Forward	TCCCTTTTAAAATAGAAGAAA	FAM	260
		Reverse	GATTATATGAAAGGATACATG		
		Heminested	TTCACTTTGTAAATTAGAACATATC		

*Censored locus caused by poor amplification success.

### Data Analyses 

To determine the source of the parasites found in the soldiers, we treated them as a discrete population and calculated the relatedness (β-diversity) between this parasite population and those identified in persons in the DRC and Guatemala. Genetic relatedness between the 3 populations was calculated by using the Nei standard genetic distance (*G_ST_*) and the Slatkin *R_ST_* (*22*). *G_ST_* is based on the infinite alleles model, which assumes that genetic differences arise through mutations and genetic drift, and was calculated in GenAlEx v6.4 (*23*) by using the following formula

in which *p_ix_* and *p_iy_* are the frequencies of the *i*th allele in populations x and y. Slatkin *R_ST_* assumes that microsatellites evolve according to the stepwise mutation model in which novel alleles are created either by deletion or addition of a single repeated unit of microsatellite that has equal probability µ/2 in both directions (*22*). *R_ST_* was calculated in SPAGeDi v1.3 (*24*) by using the following formula

in which *S* is the average squared difference in allele size between all pairs of alleles and *Sw* is the average sum of squares of the differences in allele size within each subpopulation. Pairwise *R_ST_* comparisons were calculated by using ANOVA (a nested analysis of variance approach). Principal coordinate analysis was performed to quantify the variation between the parasite populations from the soldiers and persons in the DRC and Guatemala by using results of the pairwise *R_ST_* comparisons. Principal coordinate analysis plots were generated by using GenAlEx v6.4 (*23*). We tested for associations between the neutral microsatellite markers on chromosomes 2 and 3 by using an exact test of linkage disequilibrium that had 10,000 Monte Carlo steps in Arlequin version 3.1 (*25*). After applying Bonferroni correction for multiple comparisons, we examined p values for significance.

Because we were studying parasite populations and not hosts, each individual host could contribute >1 parasite variant to the population. Since the indices above are calculated locus by locus, haplotype construction was unnecessary. However, because haplotypes are required for entering data into GenAlEx and SPAGeDi, we created “virtual” haplotypes for mixed genotypes. If >2 alleles were detected on 1 locus, distinct haplotypes were created that differed from each other only at the locus with multiple alleles. If multiple loci contained 2 alleles, 2 distinct haplotypes were created; alleles were randomly assorted for each locus. The same was done if multiple loci contained 3 alleles. If >1 loci had 2 alleles and >1 loci had 3 alleles, we created 2 full haplotypes using random assortment as above and a third haplotype that was missing data on loci that had 2 alleles. To assess whether the arrangement of these virtual haplotypes affected the analyses, we recalculated all indices of genetic relatedness using a different assortment of virtual haplotypes.

In addition to these analyses, to investigate the geographic clustering of the parasite populations, we calculated genetic distances and created a neighbor-joining phylogenetic tree (*26*) based on the Cavalli-Sforza and Edwards chord distance model (*27*) in Populations v.1.2.31 (*28*) and visualized it in the *ape* package for R (*29*). Further, we used Fast UniFrac, which is a frequently used measure of genetic differentiation between pathogen populations, to group the parasite populations with precision derived from permutation (*30*). To determine whether the observed population splits were caused by chance alone, we used 1,000 permutations. For these analyses, we used isolates for which the majority (>50%) of the microsatellite markers were characterized.

## Results

Initially, 8 microsatellite loci were characterized by capillary electrophoresis. One locus was censored in all analyses because of poor amplification success (<50%). For the remaining 7 loci, 71%–85% of the samples were successfully amplified. Seven infected soldiers and 8 infected persons from the DRC were excluded from further analyses because we were unable to amplify the majority of the microsatellites in specimens collected from them. Final analysis was conducted on samples from 5 infected soldiers, 74 infected persons from the DRC, and 40 infected persons from Guatemala.

Similar to the organisms causing infections documented in persons in the DRC, most of the organisms associated with the soldiers’ infections contained polyclonal antibodies. Of the remaining 5 infected soldiers, samples from 3 (60%) contained >1 locus that had 3 genetically distinct alleles. This is similar to the distribution in the DRC, where strains from 47.3% (n = 35) of the subjects had >2 alleles in >1 locus. All the samples from persons living in Guatemala contained a single genotype. No significant linkage disequilibrium was observed between the neutral microsatellite markers used in all 3 study populations (data not shown).

**Figure 2:**
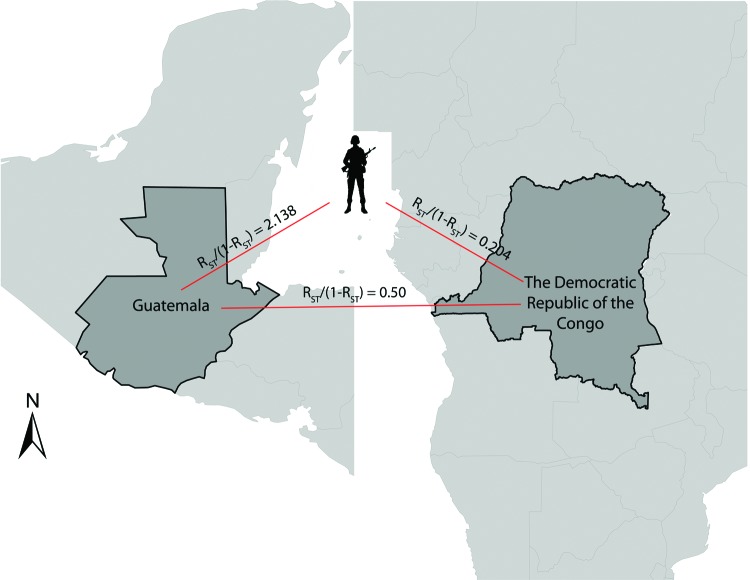
Genetic relatedness (pairwise *R_ST_* comparisons) among *Plasmodium falciparum* identified in samples from the soldiers from Guatemala, persons in the Democratic Republic of the Congo, and persons in Guatemala.

To identify the source of the infections, we measured the genetic relatedness between parasite populations obtained from the soldiers, from the DRC residents, and from residents of Guatemala. Pairwise *R_ST_*/(1–*R_ST_*) and *G_ST_* comparisons were calculated; values of ≤0 signify virtual identity whereas increasing values signify increasing divergence. Overall, parasites in samples from the soldiers were much more closely related to the parasites from the DRC ((*R_ST_*/(1 – *R_ST_*) = 0.204 and *G_ST_* = 0.278) than to parasites from Guatemala ((*R_ST_*/(1 – *R_ST_*) = 2.138 and *G_ST_* = 2.028) (Figure 2). Both metrics (*R_ST_* and *G_ST_*) indicated that parasites identified in the soldiers were more closely related to those found in the DRC than in those from Guatemala. However, pairwise *R_ST_* may be more appropriate because of the high mutation rates in microsatellites (*19*,*22*). When we used a different assortment of virtual haplotypes, genetic relatedness between the different parasite populations remained the same (data not shown).

**Figure 3:**
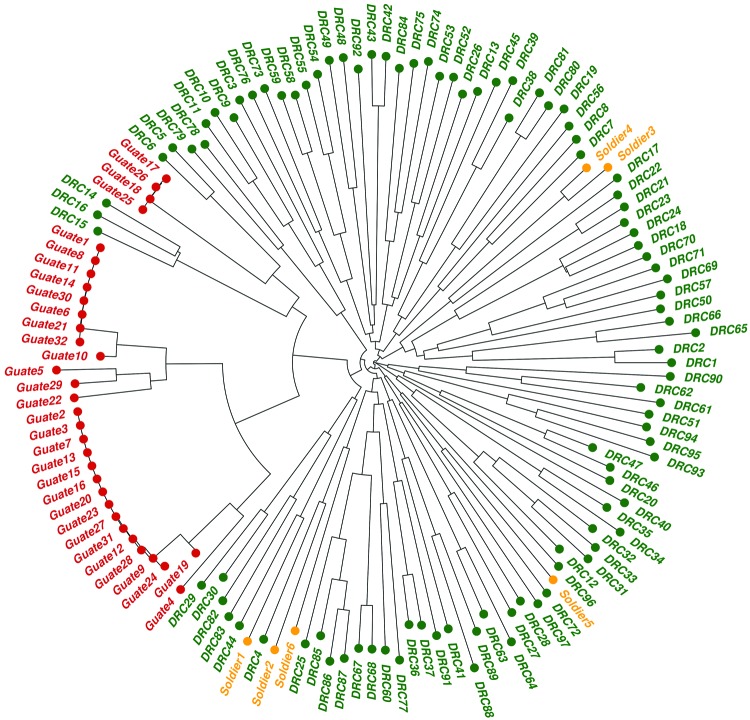
Neighbor-joining tree of 3 *Plasmodium falciparum* populations. Prefixes of genomes indicate parasite origins: Green text indicates parasite populations from the Democratic Republic of the Congo (DRC); orange indicates parasite populations detected in soldiers who were returning from the DRC to Guatemala; red indicates parasite populations from Guatemala.

**Figure 4:**
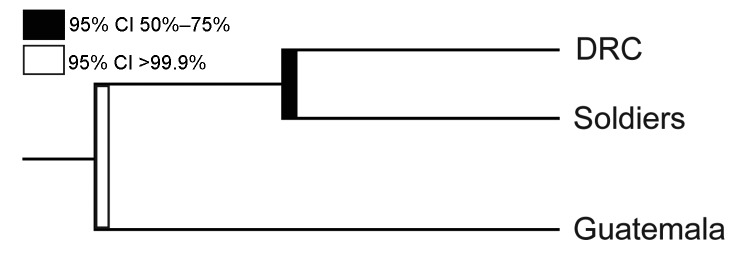
Phylogenetic tree showing predicted clustering between *Plasmodium falciparum* populations from the Democratic Republic of Congo (DRC), soldiers returning to Guatemala from the DRC, and Guatemala. The predicted split between parasites identified in samples taken in Guatemala and parasites from DRC among soldiers was significant (95% CI >99.9%) (black bar); the predicted split between parasites from DRC and returning soldiers was not significant (95% CI 50%–70%) (white bar). Computed by using Fast UniFrac (*30*) with jacknifing and 1,000 permutations.

The phylogenetic analyses also revealed a stark clustering effect between the 3 parasite populations. Visual inspection of the neighbor-joining tree (Figure 3) showed that the parasites found in the soldiers were part of the parasite population from the DRC and the parasites found in Guatemala were distinct from the other 2 populations. The ecologic clustering algorithm, after 1,000 jackknife permutations, also clustered the parasites from the soldiers with the parasite population in the DRC while they remained distinct from the parasites from Guatemala (Figure 4). The predicted differences between the parasites from Guatemala and those from the DRC and soldiers returning to Guatemala were statistically significant (>99.9% confidence).

We further attempted to examine the genetic relatedness of parasites, comparing those from each DRC cluster, the soldiers, and Guatemala. We observed that the parasites identified in samples from soldiers were more closely related to every selected DHS cluster within the DRC than to parasites from the samples from persons living in Guatemala (*R_ST_*/(1 – *R_ST_*) range = 0.065–0.202) (data not shown).

## Discussion

Molecular tools have become valuable in tracking the source of infectious agents in outbreak investigations (*31*,*32*). This study, which was based on the use of population genetic analyses of microsatellite data, supports previous epidemiologic findings that an outbreak of *P. falciparum* malaria in soldiers from Guatemala, who returned after their peacekeeping mission in the DRC in 2010, was caused by an imported parasite population from the DRC (*9*). This study further validates the use of molecular epidemiologic tools in malaria outbreak investigations.

Genetic relatedness among 3 parasite populations was first assessed by pairwise *R_ST_* and G_ST_. Parasites from the soldiers were more closely related to those from the DRC than those from Guatemala (Figure 2). These results were corroborated by neighbor-joining phylogenetic analyses and by Fast UniFrac, which showed with >99.9% confidence that the parasites from the soldiers were part of the parasite population from the DRC and distinct from the native parasites from Guatemala (Figures 3, 4). An alternate hypothesis is that there was a prior introduction of DRC parasites into Guatemala; support for this hypothesis is provide by our neighbor-joining phylogenetic tree, which includes a subset of DRC isolates that clustered with the parasites from Guatemala (Figure 3), and that finding suggests that the soldiers were infected after returning to Guatemala. However, this subset of DRC parasites was substantially different from the dominant clones in circulation in Guatemala, and the ecologic clustering algorithm consistently clustered parasites separately (Figures 3, 4). Additionally, given the high degree of heterogeneity within DRC parasites, it is reasonable to expect some overlap between a subset of parasites from the DRC and other parasite populations. Overall, the results from the molecular analyses, as well as the epidemiologic investigation (*9*) indicate that the source of the parasites among the soldiers from Guatemala was most likely the DRC. The results support the clinical information obtained from the soldiers. None of the soldiers adhered to malaria prophylaxis or used insecticide-treated bed nets as recommended during their time in the DRC. Lack of adherence to preventive measures has been identified as a risk factor for malaria infection among travelers to malaria-endemic countries (*33*). Further, the results are consistent with the travel history of the soldiers and the hypothesis from the epidemiologic investigation that the soldiers acquired malaria while traveling through the northern DRC and that the source of the outbreak was in the DRC and not in Guatemala (*9*).

Countries in Central America are experiencing low levels of malaria incidence, and several of them are taking steps toward its elimination (*4*,*34*). In June 2013, the Council of Health Ministers from Central America and the Dominican Republic called for malaria elimination in the region by 2020 (*35*). Although chloroquine-resistant *P. falciparum* strains are widespread, Central America is one of the few regions in the world where chloroquine remains an effective treatment option for locally acquired malaria (*6*–*8*). Importation of chloroquine-resistant strains could lead to increased malaria-related illness and deaths, even though the local *Anopheles* spp. vector population may be refractory to foreign *Plasmodium* strains (*36*,*37*). Of the 12 soldiers who died after returning from the peacekeeping mission, 1 was found to have been infected with parasites of the *P. falciparum* chloroquine-resistance transporter (*pfcrt*) genotype CVIET, but it was not clear whether this patient acquired resistant parasites from the DRC or locally (*9*). It is unlikely that the patient acquired chloroquine-resistant parasites locally because these parasites are absent in Central America (*7*,*8*). 

If chloroquine-resistant strains circulated in Guatemala, chloroquine drug pressure would positively select them and ultimately render chloroquine ineffective. Conversely, chloroquine resistance has been widespread in the DRC since at least 2002, which is supported by the report that the hallmark K76T mutation has been found in 93% of Congolese specimens (*38*). The current data provide evidence that this soldier acquired chloroquine-resistant *P. falciparum* in the DRC. Indeed, the microsatellite data suggested that this soldier had multiple strains. Given increasing globalization, international travel, and high frequency of military peacekeeping missions from Central America to Africa, persons returning from a malaria-endemic country who have malarial symptoms should be suspected of harboring chloroquine-resistant strains. These circumstances also provide impetus for countries such as Guatemala to make artemisinin-based combination therapies and other appropriate treatment available to treat imported multidrug-resistant malaria cases.

This study had several limitations. First, we included samples from only 7 DHS clusters. Including more clusters, especially those near Bunia, Isiro, and Dungo, which the soldiers reported having traveled through, would have helped in triangulating the source of infection with a higher resolution. Also, the local samples collected in Guatemala included in the study were collected during different years (1998–2000) from the outbreak. Because of the low number of malaria cases reported annually in Guatemala (31 total cases reported in 2010), it was not feasible to include samples from persons in Guatemala that were collected closer to the time of the outbreak. Further, of the 12 patients identified during the outbreak investigation, we were able to successfully characterize microsatellite profiles for *P. falciparum* parasites in only 5 patients who had microscopically detectable parasitemia levels. Microsatellites could not be amplified in samples obtained from any of the 7 patients who had asymptomatic *P. falciparum* infection. A possible explanation for this failure could be the potential degradation of gDNA between the time of extraction and time at which the microsatellite markers were characterized. However, the results of the study were likely unaffected as shown by the stark similarity between parasites from the soldiers and persons in the DRC and substantial divergence between parasites from the soldiers and persons in Guatemala.

Molecular epidemiology and population genetic tools have been used successfully to identify geographic areas where outbreaks of poliomyelitis originate, leading to focused intensification of public health efforts to reduce polio-related illness and future outbreaks (*39*,*40*). Here we have demonstrated the use of molecular tools to conclusively identify the source of a parasite population during an outbreak investigation. Because of the extreme minimal diversity of the parasites from Guatemala, we were able to triangulate the source of the outbreak with high statistical significance. However, if each of the 3 parasite populations were as highly diverse as those in the DRC, we would require a greater number of samples and would use other genotyping methods which characterize parasite subpopulations on a finer scale, such as next-generation deep-sequencing methods to sequence polymorphic targets or whole genomes. There is also a clear need for capacity strengthening of sample collection, storage, and extraction techniques to support genotyping in countries that report low malaria transmission rates. These molecular tools could help strengthen existing surveillance efforts to prevent future outbreaks and the reintroduction of malaria in countries working toward malaria elimination.
